# Supplementary material for: Child stature, maternal education, and early childhood development in Nigeria
Source: PLoS One. 2021 Dec 23;16(12):e0260937. doi: 10.1371/journal.pone.0260937 (PMC8700053; doi:10.1371/journal.pone.0260937)
Supplement: S1 Appendix — (DOCX) [file pone.0260937.s001.docx]

## S1 Text: The steps involved in the Double Machine Learning (DML) or cross-fit partialing-out approach^[[1]](#footnote-1)^

1. Divide the data in roughly equal-sized randomly selected subsamples 1 and 2.

2. In sample 1 (subscript 1 denotes estimates from sample 1):

a. Run a LASSO of $D$ on $Z$. Let $\tilde{Z}_{D_{1}}$ be the covariates selected.

b. Regress $D$ on $\tilde{Z}_{D_{1}}$. Let $\hat{\gamma}_{1}$ be the estimated coefficients.

c. Run a LASSO of $Y$ on $Z$. Let $\tilde{Z}_{Y_{1}}$ be the covariates selected.

d. Regress $Y$ on $\tilde{Z}_{Y_{1}}$. Let $\hat{\delta}_{1}$ be the estimated coefficients.

3. In sample 2: use the coefficients and the individual values of the covariates selected from the above step in sample 1 to construct the “residualized” or “partialed-out” $\tilde{D}$ and $\tilde{Y}$ as follows

a. Fill in $\tilde{D}{=D-\tilde{Z}}_{D_{1}}\hat{\gamma}_{1}$

b. Fill in $\tilde{Y}{=Y-\tilde{Z}}_{Y_{1}}\hat{\delta}_{1}$

Note: the values of $\tilde{D}$ and $\tilde{Y}$ are filled in only for individual observations in sample 2, and they are left missing for the individual observations in sample 1 (but they get filled in step 5 below)

4. Still in sample 2 (subscript 2 denotes estimates from sample 2):

a. Run a LASSO of $D$ on $Z$. Let $\tilde{Z}_{D_{2}}$ be the covariates selected.

b. Regress $D$ on $\tilde{Z}_{D_{2}}$. Let $\hat{\gamma}_{2}$ be the estimated coefficients.

c. Run a LASSO of $Y$ on $Z$. Let $\tilde{Z}_{Y_{2}}$ be the covariates selected.

d. Regress $Y$ on $\tilde{Z}_{Y_{2}}$. Let $\hat{\delta}_{2}$ be the estimated coefficients.

5. In sample 1: use the coefficients and the covariates selected from the above step in sample 2 to construct the “residualized” $\tilde{D}$ and $\tilde{Y}$ as follows

a. Fill in $\tilde{D}{=D-\tilde{Z}}_{D_{2}}\hat{\gamma}_{2}$

b. Fill in $\tilde{Y}{=Y-\tilde{Z}}_{Y_{2}}\hat{\delta}_{2}$

6. In the full sample: Regress $\tilde{Y}$ on $\tilde{D}$. The estimate of$\beta$ from the preceding regression and its test statistics are then the coefficient on $\tilde{D}$ and its test statistics.

The splitting of the sample and the fact that coefficients are obtained from one sample and used in an independent sample is something that adds robustness to the DML approach. In the algorithm presented above, the full sample is split in two. In fact, the estimates presented are based on splitting the sample randomly into 10 parts and this 10-part random splitting is repeated 10 times and results averaged (the commands used were *xporegress* and *xpologit* for continuous and binary dependent variables, respectively, with the options *select (cv)* and *resample (10)* in Stata v.16).

1. Adapted from “Lasso inference intro” from Stata Lasso Reference Manual, Release 16. [↑](#footnote-ref-1)
