# Supplementary material for: Child stature, maternal education, and early childhood development in Nigeria
Source: PLoS One. 2021 Dec 23;16(12):e0260937. doi: 10.1371/journal.pone.0260937 (PMC8700053; doi:10.1371/journal.pone.0260937)
Supplement: S1 Table — (DOCX) [file pone.0260937.s002.docx]

## S1 Table: Child, Mother and Household characteristics

| Variable | Urban | Rural | North- Central | North- East | North- West | South- East | South- South | South- West |
| --- | --- | --- | --- | --- | --- | --- | --- | --- |
| Height-for-age | -1.275 | -1.943 | -1.466 | -2.058 | -2.258 | -0.692 | -0.770 | -0.919 |
| Stunted | 0.305 | 0.495 | 0.349 | 0.527 | 0.584 | 0.172 | 0.187 | 0.190 |
| Underweight | 0.230 | 0.354 | 0.196 | 0.402 | 0.426 | 0.140 | 0.135 | 0.163 |
| Wasted | 0.228 | 0.351 | 0.194 | 0.398 | 0.422 | 0.139 | 0.134 | 0.162 |
| Child is girl | 0.493 | 0.487 | 0.482 | 0.500 | 0.488 | 0.479 | 0.489 | 0.487 |
| Child’s age | 47.39 | 47.03 | 47.26 | 47.26 | 47.02 | 46.74 | 47.22 | 47.22 |
| Child's birth order (1st, 2/3rd, 4/6th | 2.303 | 2.402 | 2.265 | 2.478 | 2.509 | 2.180 | 2.150 | 2.071 |
| Child is the child of the head of household | 0.955 | 0.955 | 0.956 | 0.965 | 0.969 | 0.895 | 0.902 | 0.954 |
| Mother's years of education | 7.583 | 3.114 | 4.852 | 2.644 | 2.326 | 9.983 | 9.280 | 9.554 |
| Mother's age at birth | 27.230 | 26.377 | 26.456 | 26.273 | 26.004 | 28.380 | 27.730 | 28.378 |
| Mother in a union | 0.944 | 0.965 | 0.968 | 0.960 | 0.977 | 0.924 | 0.888 | 0.948 |
| Mother consumes media | 0.766 | 0.436 | 0.411 | 0.389 | 0.592 | 0.611 | 0.669 | 0.795 |
| Mother believes wife beating ok | 0.277 | 0.410 | 0.458 | 0.315 | 0.397 | 0.284 | 0.394 | 0.261 |
| Education category of household head | 3.219 | 2.897 | 2.672 | 2.699 | 3.486 | 2.504 | 2.752 | 2.837 |
| Household size | 8.255 | 8.771 | 8.434 | 9.596 | 9.694 | 6.322 | 6.245 | 5.792 |
| Dependency ratio | 1.497 | 1.672 | 1.536 | 1.699 | 1.710 | 1.581 | 1.449 | 1.372 |
| Under 14 /adults | 1.463 | 1.625 | 1.497 | 1.666 | 1.666 | 1.492 | 1.405 | 1.336 |
| Over 65 / adults | 0.034 | 0.047 | 0.039 | 0.033 | 0.045 | 0.089 | 0.044 | 0.036 |
| Rooms per capita | 0.353 | 0.375 | 0.411 | 0.353 | 0.349 | 0.422 | 0.395 | 0.349 |
| Number of: |  |  |  |  |  |  |  |  |
| Cattle, milk cows, or bulls | 0.382 | 2.570 | 2.143 | 2.778 | 2.234 | 0.097 | 0.240 | 0.465 |
| Horses, donkeys, or mules | 0.069 | 0.145 | 0.022 | 0.115 | 0.216 | 0.038 | 0.048 | 0.046 |
| Goats | 1.078 | 4.428 | 3.775 | 4.239 | 4.391 | 0.919 | 0.419 | 0.750 |
| Sheep | 0.654 | 2.670 | 1.565 | 2.488 | 3.125 | 0.194 | 0.125 | 0.306 |
| Chickens | 2.795 | 9.195 | 10.586 | 6.834 | 8.425 | 4.408 | 2.638 | 2.935 |
| Pigs | 0.156 | 0.351 | 0.796 | 0.177 | 0.164 | 0.259 | 0.213 | 0.249 |
| Camels | 0.037 | 0.071 | 0.014 | 0.142 | 0.054 | 0.029 | 0.037 | 0.014 |
| Duck | 0.134 | 0.714 | 1.199 | 0.726 | 0.424 | 0.048 | 0.039 | 0.063 |
| Geese | 0.055 | 0.083 | 0.120 | 0.053 | 0.095 | 0.014 | 0.046 | 0.028 |
| Quail | 0.033 | 0.103 | 0.022 | 0.047 | 0.164 | 0.038 | 0.049 | 0.002 |
| Cultured Fish | 0.186 | 0.195 | 0.415 | 0.033 | 0.096 | 0.126 | 0.450 | 0.367 |
| Number of mosquito nets | 2.009 | 2.445 | 1.978 | 2.799 | 2.764 | 1.386 | 1.431 | 1.269 |
| Main material of roof | 1.941 | 1.490 | 1.636 | 1.496 | 1.529 | 1.919 | 1.917 | 1.922 |
| Main material of wall | 1.816 | 1.151 | 1.420 | 1.100 | 1.186 | 1.826 | 1.772 | 1.890 |
| Main material of floor | 1.735 | 1.009 | 1.424 | 1.046 | 0.938 | 1.698 | 1.712 | 1.826 |
| Handwashing station avail. | 0.203 | 0.082 | 0.089 | 0.141 | 0.116 | 0.101 | 0.141 | 0.132 |
| Improved water | 0.684 | 0.517 | 0.527 | 0.506 | 0.558 | 0.713 | 0.682 | 0.652 |
| Improved sanitary facility | 0.489 | 0.284 | 0.257 | 0.385 | 0.373 | 0.404 | 0.354 | 0.289 |
| Household: open defecation | 0.054 | 0.321 | 0.523 | 0.183 | 0.147 | 0.238 | 0.218 | 0.247 |
| Has agricultural land | 0.399 | 0.856 | 0.770 | 0.715 | 0.813 | 0.663 | 0.608 | 0.359 |
| Household has electricity | 0.845 | 0.321 | 0.440 | 0.408 | 0.402 | 0.654 | 0.679 | 0.796 |
| Household owns: |  |  |  |  |  |  |  |  |
| Radio | 0.700 | 0.585 | 0.610 | 0.580 | 0.619 | 0.699 | 0.607 | 0.702 |
| Television | 0.742 | 0.278 | 0.472 | 0.307 | 0.265 | 0.694 | 0.725 | 0.799 |
| Non-mobile phone | 0.040 | 0.024 | 0.023 | 0.024 | 0.041 | 0.011 | 0.023 | 0.019 |
| Refrigerator | 0.412 | 0.093 | 0.188 | 0.102 | 0.134 | 0.327 | 0.384 | 0.398 |
| VCR, VCD, DVD | 0.657 | 0.247 | 0.418 | 0.251 | 0.236 | 0.641 | 0.662 | 0.727 |
| Sewing machine | 0.220 | 0.141 | 0.136 | 0.175 | 0.193 | 0.117 | 0.135 | 0.139 |
| Clock | 0.700 | 0.414 | 0.537 | 0.387 | 0.415 | 0.677 | 0.744 | 0.749 |
| Generator | 0.394 | 0.186 | 0.301 | 0.131 | 0.130 | 0.536 | 0.503 | 0.531 |
| Computer | 0.123 | 0.025 | 0.060 | 0.030 | 0.047 | 0.073 | 0.083 | 0.107 |
| Water heater | 0.192 | 0.046 | 0.086 | 0.060 | 0.090 | 0.070 | 0.146 | 0.145 |
| Watch | 0.721 | 0.471 | 0.502 | 0.519 | 0.463 | 0.707 | 0.749 | 0.770 |
| Mobile phone | 0.914 | 0.675 | 0.821 | 0.745 | 0.622 | 0.875 | 0.886 | 0.938 |
| Bicycle | 0.222 | 0.297 | 0.197 | 0.345 | 0.336 | 0.251 | 0.200 | 0.077 |
| Motorcycle or scooter | 0.292 | 0.448 | 0.547 | 0.269 | 0.464 | 0.372 | 0.344 | 0.266 |
| Animal-drawn cart | 0.030 | 0.134 | 0.017 | 0.149 | 0.168 | 0.018 | 0.009 | 0.004 |
| Car / truck | 0.212 | 0.077 | 0.139 | 0.076 | 0.103 | 0.165 | 0.126 | 0.211 |
| Boat with motor | 0.013 | 0.023 | 0.029 | 0.014 | 0.013 | 0.016 | 0.055 | 0.017 |
| Tricycle (Keke Napep) | 0.033 | 0.013 | 0.013 | 0.017 | 0.024 | 0.029 | 0.018 | 0.011 |
| Household owns house | 0.530 | 0.870 | 0.801 | 0.802 | 0.898 | 0.665 | 0.570 | 0.331 |
| Household has bank account | 0.654 | 0.247 | 0.390 | 0.280 | 0.245 | 0.643 | 0.598 | 0.712 |
