# Supplementary material for: Child stature, maternal education, and early childhood development in Nigeria
Source: PLoS One. 2021 Dec 23;16(12):e0260937. doi: 10.1371/journal.pone.0260937 (PMC8700053; doi:10.1371/journal.pone.0260937)
Supplement: S2 Table — (DOCX) [file pone.0260937.s003.docx]

## S2 Tables of DML results by zone

**Table 1: North Central: Early child development outcomes**

|  | **Stunt** | | **Mother's education** | |
| --- | --- | --- | --- | --- |
|  | **Coeff** | **p-value** | **Coeff** | **p-value** |
| **ECDI** | -0.134 | 0.004 | 0.021 | 0.001 |
|  | **OR** | **p-value** | **OR** | **p-value** |
| **ECD UNICEF** | 0.808 | 0.075 | 1.040 | 0.018 |
| **Literacy and numeracy** | 0.562 | 0.002 | 1.071 | 0.000 |
| **Physical development** | 0.777 | 0.190 | 1.002 | 0.936 |
| **Learning** | 0.614 | 0.001 | 1.031 | 0.114 |
| **Socio-emotional** | 1.160 | 0.226 | 0.995 | 0.725 |

**Table 2: North East: Early child development outcomes**

|  | **Stunting** | | **Mother's education** | |
| --- | --- | --- | --- | --- |
|  | **Coeff** | **p-value** | **Coeff** | **p-value** |
| **ECDI** | -0.189 | 0.000 | 0.032 | 0.000 |
|  | **OR** | **p-value** | **OR** | **p-value** |
| **ECD UNICEF** | 0.596 | 0.000 | 1.057 | 0.006 |
| **Literacy and numeracy** | 0.480 | 0.000 | 1.129 | 0.000 |
| **Physical development** | 0.671 | 0.043 | 1.121 | 0.001 |
| **Learning** | 0.820 | 0.176 | 1.058 | 0.024 |
| **Socio-emotional** | 0.837 | 0.162 | 0.974 | 0.171 |

**Table 3: North West: Early child development outcomes**

|  | **Stunting** | | **Mother's education** | |
| --- | --- | --- | --- | --- |
|  | **Coeff** | **p-value** | **Coeff** | **p-value** |
| **ECDI** | -0.167 | 0.000 | 0.022 | 0.000 |
|  | **OR** | **p-value** | **OR** | **p-value** |
| **ECD UNICEF** | 0.790 | 0.012 | 1.026 | 0.070 |
| **Literacy and numeracy** | 0.644 | 0.003 | 1.040 | 0.088 |
| **Physical development** | 0.675 | 0.030 | 1.053 | 0.061 |
| **Learning** | 0.695 | 0.001 | 1.017 | 0.240 |
| **Socio-emotional** | 1.024 | 0.805 | 1.015 | 0.306 |

**Table 4: South East: Early child development outcomes**

|  | **Stunting** | | **Mother's education** | |
| --- | --- | --- | --- | --- |
|  | **Coeff** | **p-value** | **Coeff** | **p-value** |
| **ECDI** | -0.223 | 0.015 | 0.014 | 0.165 |
|  | **OR** | **p-value** | **OR** | **p-value** |
| **ECD UNICEF** | 0.715 | 0.187 | 1.011 | 0.706 |
| **Literacy and numeracy** | 0.517 | 0.011 | 1.075 | 0.016 |
| **Physical development** | 0.509 | 0.046 | 1.034 | 0.440 |
| **Learning** | 0.872 | 0.530 | 1.013 | 0.547 |
| **Socio-emotional** | 1.172 | 0.556 | 0.945 | 0.045 |

**Table 5: South South: Early child development outcomes**

|  | **Stunt** | | **Mother's education** | |
| --- | --- | --- | --- | --- |
|  | **Coeff** | **p-value** | **Coeff** | **p-value** |
| **ECDI** | -0.328 | 0.000 | 0.002 | 0.848 |
|  | **OR** | **p-value** | **OR** | **p-value** |
| **ECD UNICEF** | 0.453 | 0.000 | 0.980 | 0.377 |
| **Literacy and numeracy** | 0.397 | 0.000 | 1.048 | 0.070 |
| **Physical development** | 0.640 | 0.172 | 1.020 | 0.670 |
| **Learning** | 0.458 | 0.000 | 0.980 | 0.401 |
| **Socio-emotional** | 0.852 | 0.358 | 0.951 | 0.030 |

**Table 6: South West: Early child development outcomes**

|  | **Stunt** | | **Mother's education** | |
| --- | --- | --- | --- | --- |
|  | **Coeff** | **p-value** | **Coeff** | **p-value** |
| **ECDI** | -0.277 | 0.000 | 0.018 | 0.056 |
|  | **OR** | **p-value** | **OR** | **p-value** |
| **ECD UNICEF** | 0.491 | 0.001 | 1.002 | 0.950 |
| **Literacy and numeracy** | 0.519 | 0.001 | 1.069 | 0.020 |
| **Physical development** | 1.046 | 0.892 | 1.066 | 0.119 |
| **Learning** | 0.607 | 0.010 | 0.996 | 0.813 |
| **Socio-emotional** | 0.564 | 0.006 | 0.994 | 0.843 |
