# Supplementary material for: Child stature, maternal education, and early childhood development in Nigeria
Source: PLoS One. 2021 Dec 23;16(12):e0260937. doi: 10.1371/journal.pone.0260937 (PMC8700053; doi:10.1371/journal.pone.0260937)
Supplement: Attachment — Submitted filename: PONE review AA.docx [file pone.0260937.s004.docx]

The paper addresses the issue of efficacy of the Randomized controlled trials[ called ad-hoc approach in the paper] vis a vis a direct machine learning[ DML] approach where using artificial intelligence, controls can be assigned across different variables

The results of the two approaches are interesting, and sometimes conflicting. One example being that in both rural and urban areas, the DML approach suggests that mother education has no impact on a child being on track in learning, whereas the ad-hoc approach suggests that it does. Similarly, mother’s education impact on a child’s chronic malnutrition status measured by whether the child is stunted differs in both methods in rural and urban areas.

There are two basic issues here. One is from the policy perspective- in the face of conflicting results, which one should the policy maker rely on? Often the data quality and its limitations in all their granularity are not know at the top levels of decision making. The interventions would differ widely with two starkly different pieces of evidence based on the same set of people. This contradiction needs to be addressed by the authors. The paper may delve deeper into the faithful adherence to reality and the offering for the policy makers for both approaches and have a clear set of pros and cons for each, which can be taken into account for decision making.

Secondly, RCTs are designed to eliminate biases in allocation of samples to control and intervention groups. At the same time, AI is not free of biases and in fact may “internalize” them based on the data sets. This is an ethical limitation that may skew the interpretation. The authors may address this issue as well, looking into the bias replication by AI.

Overall, with these caveats , it is an interesting and novel study, and can contribute to furthering and honing the filed of data analysis and interpretation.
