# Supplementary material for: Child stature, maternal education, and early childhood development in Nigeria
Source: PLoS One. 2021 Dec 23;16(12):e0260937. doi: 10.1371/journal.pone.0260937 (PMC8700053; doi:10.1371/journal.pone.0260937)
Supplement: Attachment — Submitted filename: Response to Reviewers.docx [file pone.0260937.s006.docx]

July 27, 2021

Dear Editors

Attached please find the revised version of our paper titled “Child Stature, Maternal Education, and Early Childhood Development in Nigeria”. We are re-submitting this revised version of our paper for consideration for publication in PLoS ONE.

In revising our paper we have taken into consideration all the comments received from the reviewers including, adhering to the submission standards, of the journal (reviewer 3), the use of a more simplified language so we can reach out more wider audience (Reviewer 2), and a more clear definition of stunting or being stunted (reviewer 4). We have not addressed some of the points of reviewer 2 as we think they are based on a misunderstanding of what this paper does. For example, reviewer 2 prefaces his comments with the statement: ”*The paper addresses the issue of efficacy of the Randomized controlled trials[ called ad-hoc approach in the paper] vis a vis a direct machine learning[ DML] approach where using artificial intelligence, controls can be assigned across different variables*”

Our paper does not address the issue of efficacy of RCTs. It simply states that RCTs are not possible to implement to address many policy relevant questions such as the one in the paper: “Does mother’s education reduced child stunting and is child stunting associated with lower ECD?” The ad-hoc approach we refer to is the ad-hoc way of choosing controls in a regression-based approach intended to examine the relation (ideally the casual relation) between stunting, mother’s education and ECD.

We look forward to hearing your decision regarding the publication of this paper

Thank you in advance for your consideration

Sincerely,

Emmanuel Skoufias

Lead Economist

Poverty and Equity Global Practice

The World Bank Washington DC USA
