# Supplementary material for: Child stature, maternal education, and early childhood development in Nigeria
Source: PLoS One. 2021 Dec 23;16(12):e0260937. doi: 10.1371/journal.pone.0260937 (PMC8700053; doi:10.1371/journal.pone.0260937)
Supplement: Attachment — Submitted filename: Reviewer comments .docx [file pone.0260937.s005.docx]

Reviewer comments on the manuscript with title “Child Stature, Maternal Education, and Early Childhood Development in

Nigeria” The comments are below

The author did not read the guidelines for article submission. The paper did not meet the criteria for publication in this journal.

1. There no line numbering

2. Document has footnotes

3. Referencing is not Vancouver style

4. There was no cover letter

5. Abstract contain abbreviation

6. There was no ethical clearance

**ABSTRACT**: The abstract is very difficult to understand and has no bearing on the kind of research that was done.

**INTRODUCTION**: This is completely confusing and has no direction or thought to follow.

**METHODS**

Not structured

Looks like a literature review

Difficult to follow and understand what is being talked about.

**RESULT**:

Made no sense

The tables do not carry any information

**DISCUSSION:** The results was not discussed

**CONCLUSION**: Not applicable
